# A comparison of four different models of acute respiratory distress syndrome in sheep

**DOI:** 10.1186/s12931-020-01475-0

**Published:** 2020-08-08

**Authors:** Monique Engel, Relana M. E. Nowacki, Elly M. Jonker, Daan Ophelders, Maria Nikiforou, Nico Kloosterboer, Luc J. I. Zimmermann, Dick A. van Waardenburg, Boris W. Kramer

**Affiliations:** 1 Department of Pediatrics, School for Oncology and Developmental Biology – GROW, Maastricht University Medical Centre (MUMC+), P. Debyelaan 25, PO Box 5800, NL-6202 AZ Maastricht, The Netherlands; 2 Department of Pediatrics, School of Nutrition and Translational Research in Metabolism – NUTRIM, Maastricht University Medical Centre (MUMC+), Maastricht, The Netherlands; 3 Department of Pediatrics, School of Mental Health and Neuroscience, Maastricht University Medical Centre (MUMC+), Maastricht, The Netherlands

**Keywords:** ARDS, Acute respiratory distress syndrome, Pulmonary, Extra-pulmonary, Lung injury, Sheep

## Abstract

**Background:**

Acute respiratory distress syndrome (ARDS) can have various causes. The study objective was to investigate whether different pathophysiologic models of ARDS would show different respiratory, cardiovascular and inflammatory outcomes.

**Methods:**

We performed a prospective, randomized study in 27 ventilated ewes inducing ARDS using three different techniques to mimic the pulmonary causes of ARDS (ARDSp): warm saline lavage (*n* = 6), intratracheal hydrochloric acid (HCl; n = 6), intratracheal albumin (*n* = 10), and one technique to mimic an extrapulmonary cause of ARDS (ARDSexp): intravenous lipopolysaccharide (LPS iv; *n* = 5). ARDS was defined when PaO_2_ was < 15 kPa (112 mmHg) when ventilated with PEEP 10 cm H_2_O and FiO_2_ = 1.0. The effects on gas exchange were investigated by calculating the oxygenation index (OI) and the ventilation efficacy index (VEI) every 30 min for a period of 4 h. Post mortem lung lavage was performed to obtain broncho-alveolar lavage fluid (BALF) to assess lung injury and inflammation. Lung injury and inflammation were assessed by measuring the total number and differentiation of leukocytes, the concentration of protein and disaturated phospholipids, and interleukine-6 and -8 in the BALF. Histology of the lung was evaluated by measuring the mean alveolar size, alveolar wall thickness and the lung injury score system by Matute-Bello et al., as markers of lung injury. The concentration of interleukin-6 was determined in plasma, as a marker of systematic inflammation.

**Results:**

The OI and VEI were most affected in the LPS iv group and thereafter the HCl group, after meeting the ARDS criteria. Diastolic blood pressure was lowest in the LPS iv group. There were no significant differences found in the total number and differentiation of leukocytes, the concentration of protein and disaturated phospholipids, or interleukin-8 in the BALF, histology of the lung and the lung injury score. IL-6 in BALF and plasma was highest in the LPS iv group, but no significant differences were found between the other groups. It took a significantly longer period of time to meet the ARDS criteria in the LPS iv group.

**Conclusions:**

The LPS model caused the most severe pulmonary and cardiovascular insufficiency. Surprisingly, there were limited significant differences in lung injury and inflammatory markers, despite the different pathophysiological models, when the clinical definition of ARDS was applied.

## Introduction

Acute respiratory distress syndrome (ARDS) is characterized by the acute onset of bilateral pulmonary infiltrates and severe hypoxemia with respiratory failure, in the absence of cardiogenic pulmonary edema [1]. The underlying causes of ARDS are various but can be generally divided in two broad categories: a direct pulmonary insult, like a pulmonary infection, or an indirect insult on the lungs, such as sepsis [2–9]. If the etiology of ARDS is restricted to damage or disease of the lung itself, it is called pulmonary ARDS (ARDSp) [2–9]. If the etiology of ARDS occurs outside the lung, as in a systematic inflammatory response, it is called extra-pulmonary ARDS (ARDSexp) [2–9]. Both types of ARDS are characterized by damage of the alveolar-capillary barrier due to diffuse alveolar damage and capillary endothelial injury [4–6]. Studies in humans have reported different pathophysiology, respiratory mechanics and morphological properties between ARDSp and ARDSexp [3–8]. In the early stages of ARDSp, damage to the alveolar cells is prevalent, whereas in ARDSexp, interstitial edema is the first indication of damage [5]. Furthermore, in broncho-alveolar lung fluid (BALF), the interleukin-8 (IL-8) concentration, a pro-inflammatory cytokine of systemic inflammation and neutrophil recruitment, is significantly higher in ARDSp than in ARDSexp patients [9].

Different animal models have been used to study the pathophysiology of pulmonary and extra-pulmonary ARDS [10–14]. However, no single animal model mimics all of the clinical features of ARDS seen in humans [4]. We therefore performed a comparative study in adult sheep, in which ARDS was induced using four different insults. ARDSp was induced by lung lavage, intratracheal administration of albumin 20%, or hydrochloric acid [10, 12–14]. We chose the lung lavage and hydrochloric acid insult as ARDSp models because these are proven models for ARDSp in literature [10, 12–14]. The intratracheal albumin 20% model has been published in neonates as a rescue therapy in meconium aspiration syndrome limiting the effect on the lung and reducing the increase of Interleukin-8 [15, 16]. But in the pathophysiology of ARDS, protein influx, like albumin, in the alveoli induces serious impairment of alveolar surfactant activity [17]. These data seem to be contradictive. Therefore, we wanted to investigate the effect of this insult on respiratory, cardiovascular and inflammatory outcomes in a naive adult model. ARDSexp was induced by intravenously administering lipopolysaccharide (LPS iv) from E.coli [10, 12, 14]. The different insults were chosen to reflect the multiple pathogeneses of ARDS [10–14]. When ARDSp was induced by lung lavage or intratracheal administration of albumin 20%, the washout and/or inactivation of surfactant was considered to be the most important mechanism of injury [10, 12, 14]. This was in contrast to the intratracheal administration of hydrochloric acid where a direct alveolar damage was induced [10, 12, 13]. The intravenous LPS administration induced a systemic inflammation with secondary endothelial injury [10, 12, 14]. We hypothesized that there would be differences in pulmonary, cardiovascular and inflammatory outcomes between the ARDSp and ARDSexp group, due to the different pathophysiology. We therefore assessed oxygenation index (OI) and ventilation efficacy index (VEI) as markers for gas exchange; inflammatory cells, and differentiation in broncho-alveolar lavage fluid (BALF) and interleukin-6 (IL-6) and interleukin-8 (IL-8) in BALF as markers for alveolar inflammation; total protein in BALF as a marker for lung injury and edema; disaturated phospholipids (DSPL) in BALF as a marker for surfactant pool size; lung histology as a marker for atelectasis and overinflation; the lung injury score by Matute-Bello et al. as a marker for lung injury and IL-6 in plasma as markers of systemic inflammation. We studied the animals for 4 h after the pre-defined clinical onset of ARDS.

## Methods

### Experimental protocol

We conducted a randomized, prospective study in sheep, using four different models of ARDS. The Animal Ethical Committee of the Maastricht University Medical Centre (MUMC+), the Netherlands, approved the study.

Pulmonary causes of ARDS were mimicked using lung lavage, intratracheal administrating of albumin 20%, or hydrochloric acid [10–14]. Extra-pulmonary ARDS was induced by administrating intravenous lipopolysaccharide (LPS) from E.coli [10, 12, 14].

Twenty-seven adult Texel ewes, age 1 year plus minus 3 months, were intravenously anesthetized with thiopental, intubated, and mechanically ventilated with a pressure-controlled ventilation (PCV) (Servo 900 ventilator, Siemens, Germany) [18, 19]. The ventilator settings consisted of a peak pressure to maintain a tidal volume (Vt) of 6–8 mL/kg, a respiratory rate of 15 breaths per minute, an inspiratory time of 50%, a positive end expiratory pressure (PEEP) of 10 cm H_2_O, and a fraction of inspired oxygen (FiO_2_) of 1.0 [18, 19]. A central venous and arterial catheter were inserted. The sheep were sedated during the experiment with intravenously administered midazolam (0.2 mg/kg/h), ketamine (8 mg/kg/h), and paralyzed with repeated boluses of pancuronium (0.1 mg/kg) if needed [18, 19]. The sheep were randomly assigned to four different groups. The sheep were selected by the animal technician, who was not involved in the experiment and the insult was assigned according the date of the research. The groups varied according to the insult by which the ARDS was induced:

1. Warm saline broncho-alveolar lavage (*n* = 6) [10, 12, 14]. In this group, a broncho-alveolar lavage was performed using 500 mL per kilogram body weight of sterile 0.9% NaCl at 39 °C. This procedure was repeated every 15 min until the arterial oxygen pressure (PaO_2_) had decreased to 15 kPa (112 mmHg) at PEEP of 10 cm H_2_O and FiO_2_ of 1.0.
2. Intratracheal albumin 20% (*n* = 10) [15, 16]. In this group, 250 mg albumin 20% per kilogram body weight was administered intratracheally through a small tube that was temporarily placed in the endotracheal tube.
3. Intratracheal hydrochloric acid (HCl) (*n* = 6) [10, 12–14]. This group was given HCl 0.5 N 1.5 mL per kilogram body weight intratracheally through a small tube that was temporarily inserted in the endotracheal tube and advanced to the carina before injection.
4. Intravenous lipopolysaccharide (LPS iv) (*n* = 5) [10, 12, 14]. This group received 75 μg per kilogram body weight LPS of *E. coli* serotype 0.5B55 (Sigma Aldrich, Amsterdam The Netherlands) intravenously every hour over the course of the experiment, until the criteria of ARDS had been met.

ARDS was pre-defined as PaO_2_ < 15 kPa (112 mmHg) when ventilated with PEEP 10 cm H_2_O and FiO_2_ 1.0 [15, 16]. The length of time that passed between the initiation of lung injury and meeting the ARDS criteria was recorded. In order to make a comparison between the groups possible, the time point at which the animals fulfilled the criteria of ARDS was taken as the starting point of the 4-h study period (T = 0).

Arterial analyses for gas exchange were performed at baseline and every 30 min thereafter for the duration of the experiment. Cardio-respiratory settings were recorded every 30 min. Plasma from blood samples were collected every 30 min, spun down and stored at − 80 °C. After 4 hours, the animals were euthanized by an intravenous injection of thiopental [18, 19]. Thoracotomy was performed and the lungs were removed. The left lung was lavaged with 0.9% NaCl and broncho-alveolar lavage fluid (BALF) was collected and analyzed for differential cell counts. The remaining volume was aliquoted and frozen for measurement of total protein, disaturated phospholipids (DSPL), and interleukin-6 (IL-6) and interleukin-8 (IL-8) at − 80 °C. The inflated right lower lobe of the lung was fixed in buffered 4% formaldehyde and samples were taken for histology and lung injury score.

### Oxygenation index (OI) and ventilation efficacy index (VEI)

As markers of gas exchange, OI and VEI were calculated from the cardio-respiratory parameters and blood gas analyses before and after lavage, and every 30 min during the 4-h study period [18, 19]. OI was calculated by multiplying the oxygen fraction times mean airway pressure (cm H_2_O) and dividing this by PaO_2_ (mm Hg) ((FiO_2_ x MAP)/PaO_2_). The VEI was calculated by dividing 3800 by the pressure difference between the PEEP and peak pressure (in cm H_2_O) times the respiratory rate (breaths/min) times the PaCO_2_ (mm Hg) (3800/(PIP-PEEP) x RR x PaCO_2_).

### Inflammatory cell count and differentiation in BALF

A cell count was performed after mixing the BALF with 0.4% trypan blue stain, (Gibco Invitrogen Corporation, California, USA) using the modified Neubauer Hemocytometer (Hirschmann EM Techcolor) [20]. A May-Grünwald-Giemsa staining was performed on the BALF smears, in which all neutrophils, lymphocytes and monocytes were counted in 200 cells. For each cell type, the total number of cells was calculated per kilogram body weight [20]. This was used as marker for inflammation.

### Total protein in BALF

The protein concentration of the BALF was measured using the Micro BCA Protein Assay Reagent Kit (Thermo Fisher Scientific Inc., Illinois, USA), according to the manufacturer’s instructions. The microtiter plate was read at a wavelength of 562 nm. All photospectro-analyses were done using Multiskan Spectrum hardware and SkanIt RE for MSS 2.2 software (Thermo Electron Corporation**,** Massachusetts**,** USA). Translation of optical densities into concentrations was carried out using GraphPad Prism 5 software (GraphPad Software, California, USA) [21, 22]. Total protein in BALF was used as a marker for lung injury and interstitial edema.

### Disaturated phospholipids in BALF

The DSPL in BALF was measured after 10 min of centrifugation of the BALF at 300×g and 4 °C, and 1 mL of the supernatant BALF was evaporated overnight at 60 °C under continuous nitrogen gas flow. The dry BALF was dissolved in a mixture of carbon tetrachloride and osmium tetroxide and disaturated phospholipids were isolated according to Mason et al. [23]. The DSPL were dissolved in chloroform and quantified according to Stewart, with some minor modifications, as described by Been et al. [24, 25]. The DSPL in BALF was taken as a marker for the surfactant pool size.

### Interleukin (IL)-6 and IL-8 in BALF; IL-6 in plasma

In the BALF and in the plasma, respectively, the concentration of IL-6 and IL-8 were quantified with a sheep-specific sandwich ELISA [21, 26] and used as markers of lung and systemic inflammation.

### Histology and lung injury score

We evaluated lung injury using histology of the right lower lobe of the lung, by measuring the mean alveolar size (MAS), alveolar wall thickness (AWT) and the lung injury score system by Matute-Bello et al. [27]. MAS was used as a marker of atelectasis and overinflation, while AWT was used as a marker for edema. The lung injury score system scored neutrophils in the alveolar space (A), neutrophils in the interstitial space (B), hyaline membranes (C), proteinaceous debris filling the airspace (D) and alveolar septal thickening (E). Every item was given a score between 0 to 2. The score was calculated by: ((20 x A) + (14 x B) + (7 x C) + (7 x D) + (2 x E))/number of fields × 100, leading to a score between zero (no lung injury) and one (severe lung injury) [27]. Samples of the lung were imbedded in paraffin, cut into 4 μm slices, deparaffinized in an ethanol series, stained with hematoxylin and eosin, dehydrated, and cover slipped. To measure MAS and AWT per location, 10 representative microscopic images were made with a 10-fold magnification using the Leica microscope (Axioskop 4.0; Zeiss) and Leica Qwin Pro version 3.4.0 software (Leica Microsystems, Mannheim, Germany). All images were uploaded and analyzed by Matlab 6.0 software (The Mathworks, Inc. Massachusetts, USA) to measure MAS and AWT in a blinded manner. To calculated the lung injury score 20 random high-power fields (400x total magnification) were scored per animal, by a blinded researcher, and the lung injury score was calculated per animal [27].

### Statistical analysis

Statistical analysis was performed using one-way ANOVA with a Bonferroni post-hoc test in Graphpad Prism. Data are presented as mean ± SEM unless individual measurements are shown; *p* < 0.05 was considered to be statistically significant.

## Results

In total, 27 sheep were included in the study. ARDS was induced in all four experimental models according to the predefined criteria (PaO2 < 15 kPa (112 mmHg), PEEP 10 cm H2O, FiO2 1.0). The respiratory and circulatory parameters of the four groups before ARDS was induced, and after the ARDS criteria had been met, are shown in Table 1. There were no differences between the groups at both time points, except for the diastolic blood pressure that was lower in the lavage group after the ARDS criteria had been met. All animals - except for one in the LPS iv group - survived the 4-h study period of the experiment (Table 2).

**Table 1:** The respiratory and circulatory parameters of the four groups before ARDS was induced and after the criteria for ARDS had been met (mean ± SD)

	N	PIP	OI	VEI	Heart rate	Systolic blood	Diastolic blood
		(cm H2O)			(beats/min)	pressure (mmHg)	pressure (mmHg)
Lavage pre	6	21 ± 2	3.2 ± 0.5	0.44 ± 0.07	117 ± 17	142 ± 6	123 ± 5
Albumin 20% pre	10	18 ± 2	2.9 ± 0.5	0.53 ± 0.11	118 ± 28	133 ± 23	113 ± 20
HCl pre	6	20 ± 3	3.6 ± 0.6	0.47 ± 0.9	99 ± 17	125 ± 20	106 ± 18
LPS iv pre	5	20 ± 3	3.4 ± 0.7	0.43 ± 0.09	112 ± 21	125 ± 10	108 ± 8
*p* < 0.05 pre		no	no	no	no	no	no
Lavage post	6	34 ± 3	45.1 ± 38.7	0.17 ± 0.05	112 ± 13	95 ± 16	63 ± 18
Albumin 20% post	10	36 ± 6	37.4 ± 12	0.16 ± 0.07	130 ± 29	119 ± 17	99 ± 19
HCl post	6	38 ± 4	36.8 ± 7.8	0.18 ± 0.03	108 ± 31	116 ± 6	99 ± 10
LPS iv post	5	36 ± 10	62.5 ± 17.7	0.11 ± 0.01	127 ± 14	106 ± 21	88 ± 22
*p* < 0.05 post		no	no	no	no	no	**yes**

**Table 2:** The characteristics and parameters at the end of the experiment, 4 h after the criteria for ARDS had been met (mean ± SD)

	Lavage	Albumin 20%	HCl	LPS	*p* < 0.05
N	6	10	6	5	
Death during exp	0	0	0	1	
Cells BALF (corr) (cells/kg)	43.7 ± 19.4	204.7 ± 509.2	38 ± 33.5	73.2 ± 53.5	no
Neutro BALF (corr) (cells/kg)	12.5 ± 8.8	58.4 ± 142.2	29.3 ± 30.1	8.2 ± 7.1	no
Protein BALF (corr) (mg/kg)	60.1 ± 41.6	89.5 ± 80.4	54.4 ± 40.8	32.3 ± 22.2	no
DSPL BALF (corr) (mg/kg)	0.087 ± 0.061	0.103 ± 0.061	0.071 ± 0.011	0. 132 ± 0.09	no
IL-8 BALF (corr) (ng/kg)	6.66 ± 5.67	4.42 ± 5.5	3.46 ± 1.82	6.95 ± 2.5	no
MAS (μm^2^)	6782 ± 1665	7078 ± 1694	7634 ± 3705	5158 ± 2400	no
AWT (μm^2^)	26.2 ± 2.1	27.8 ± 2.2	30 ± 3.8	29.4 ± 2.7	no
Lung injury score	0.025 ± 0.004	0.023 ± 0.003	0.024 ± 0.004	0.025 ± 0.003	no

**Fig. 1:**
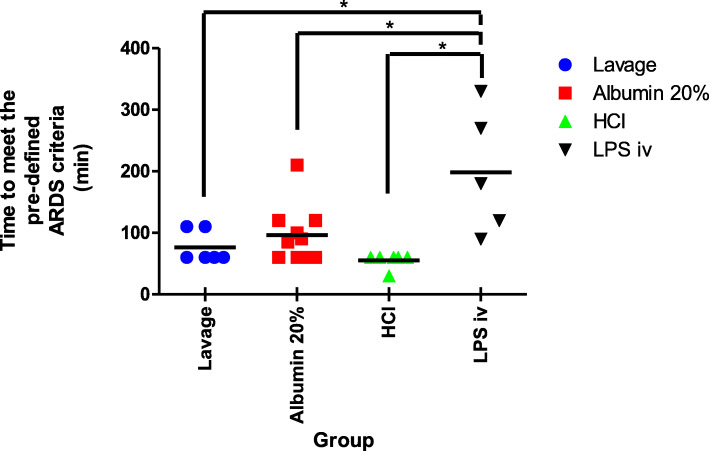
The time from the start of the experimental procedure until the pre-defined definition of ARDS had been met; PaO2 < 15 kPa when ventilated with PEEP 10 cm H2O and FiO2 1.0. Time taken to meet the pre-defined criteria for ARDS was significantly longer in the LPS iv group than the other three groups (* *p* < 0.05)

**Fig. 2:**
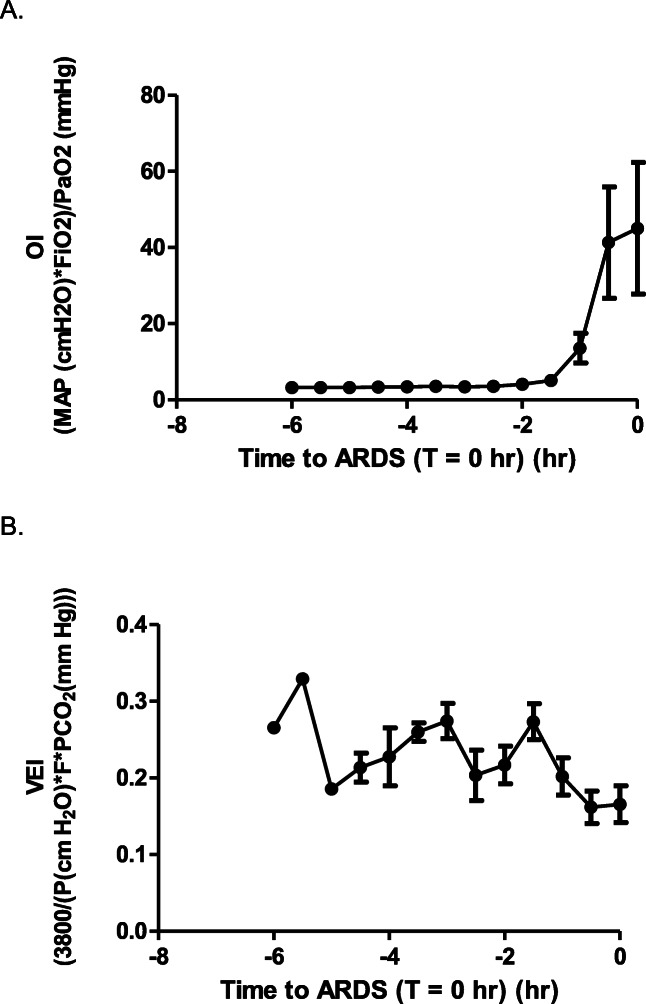
The oxygenation index (OI) (2 A) and ventilation efficacy index (VEI) (2 B) in time to reach the pre-defined definition of ARDS, which is defined as T = 0 h, in the LPS iv group, the ARDSexp group

The amount of time from the start of the experimental procedure until the pre-defined definition of ARDS had been reached was almost twice as long in the ARDSexp group as compared to the three ARDSp groups (Fig. 1). The OI and VEI in time to reach the pre-defined definition of ARDS, which was defined as T = 0 h, in the LPS iv group, the ARDSexp group, are shown in Fig. 2a and b.

### Oxygenation index (OI) and ventilation efficacy index (VEI) during the experiment

**Fig. 3:**
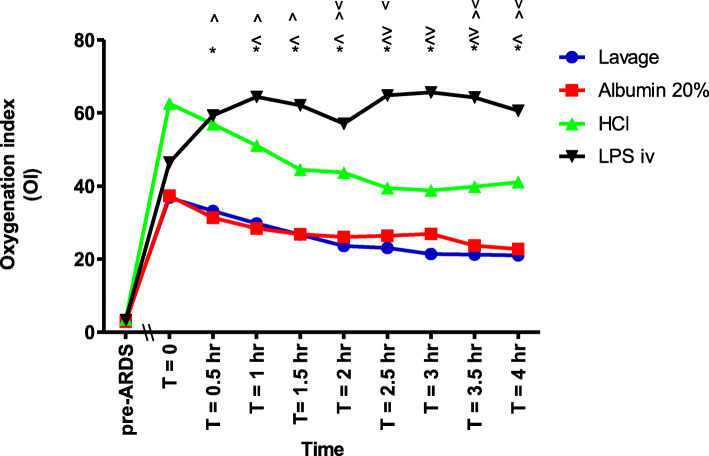
The oxygenation index (OI) between the different groups at different time points during the experiment. (*) OI LPS iv significantly higher than Albumin 20% (*p* < 0.05). (<) OI LPS iv significantly higher than lavage (*p* < 0.05). (>) OI LPS iv significantly higher than HCl (*p* < 0.05). (^) OI HCl significantly higher than Albumin 20% (*p* < 0.05). (^v^) OI HCl significantly higher than lavage (*p* < 0.05)

The OI was significantly higher, indicating more severe impairment of oxygenation, in the LPS iv group than in the albumin 20% group (Fig. 3). The OI was also significantly higher in the LPS iv group than in the lavage group, except at T = 0.5 h. Furthermore, the OI was significantly higher in the LPS iv group than in the HCl group at T = 2.5, 3 and 3.5 h (Fig. 3). The OI in the HCl group was significantly higher than in the albumin 20% group, except at T = 2.5 and 3 h. The OI in the HCI group was also significantly higher than in the lavage group, at T = 2, 2.5, 3.5 and 4 h (Fig. 3).

**Fig. 4:**
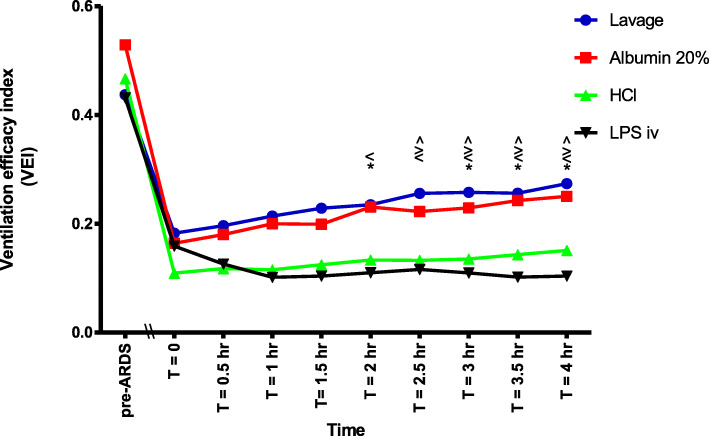
The ventilation efficacy index (VEI) between the different groups at different time points during the experiment. (*) VEI LPS iv significantly lower than Albumin 20% (*p* < 0.05). (<) VEI LPS iv significantly lower than lavage (*p* < 0.05). (>) VEI HCl significantly lower than Albumin 20% (*p* < 0.05). (^) VEI HCl significantly lower than lavage (*p* < 0.05)

The VEI was significantly lower, indicating a more severe impairment of ventilation, in the LPS iv group as compared to the lavage group from T = 1.5 h onwards. The VEI in the LPS iv group was also significantly lower than in the albumin 20% group at T = 2, 3, 3.5 and 4 h (Fig. 4). Furthermore, the VEI was significantly lower in the HCl group compared to both the albumin 20% group and the lavage group from T = 2 h onwards (Fig. 4).

The OI and VEI showed progressive improvement in the three ARDSp groups during the 4-h of the experiment (Figs. 3 and 4). This was not the case in the LPS iv group (Figs. 3 and 4).

### Blood pressure

**Fig. 5:**
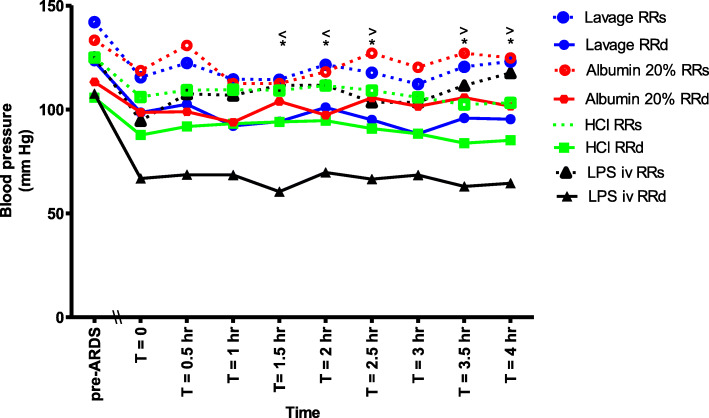
The systolic (RRs) and diastolic blood pressure (RRd) between the different groups at different time points during the experiment. (*) RRd LPS iv significantly lower than Albumin 20% (*p* < 0.05). (<) RRd LPS iv significantly lower than lavage and HCl (*p* < 0.05). (>) RRs HCl significantly lower than Albumin 20% (*p* < 0.05)

The diastolic blood pressure was significantly lower in the LPS iv group when compared to the albumin 20% group from T = 1.5 h onwards, except at T = 3 h. The diastolic blood pressure was also significantly lower in the LPS iv group compared to the HCl and lavage groups at T = 1.5 and 2 h (Fig. 5). The systolic blood pressure in the HCl group was significantly lower as compared to the albumin 20% group at T = 2.5, 3.5 and 4 h (Fig. 5).

### Cells, differentiation, total protein, disaturated phospholipids (DSPL), interleukin-6 (IL-6) and interleukin-8 (IL-8) in broncho-alveolar lavage fluid (BALF), plasma, histology and lung injury score

There were no significant differences found in the total number and differentiation of leukocytes in the BALF, which we used as a marker of inflammation. The concentrations of total protein in BALF, used as a marker of lung injury and edema, were not different. The surfactant pool size, assessed as DSPL in the BALF, was similar in all groups. There were also no significant differences between groups in the IL-8 concentration in BALF (Table 2).

**Fig. 6:**
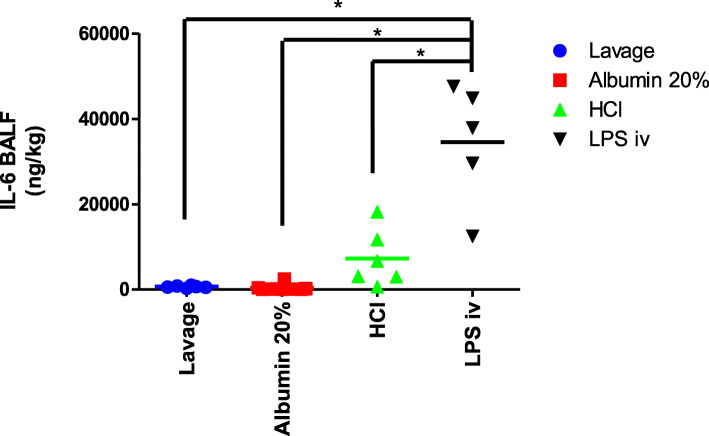
Interleukin-6 (IL-6) concentration in broncho-alveolar lavage fluid (BALF) in different groups at the end of the experiment, T = 4 h. The IL-6 concentration in BALF was significantly higher in the LPS iv group compared to the other three groups (* *p* < 0.001)

**Fig. 7:**
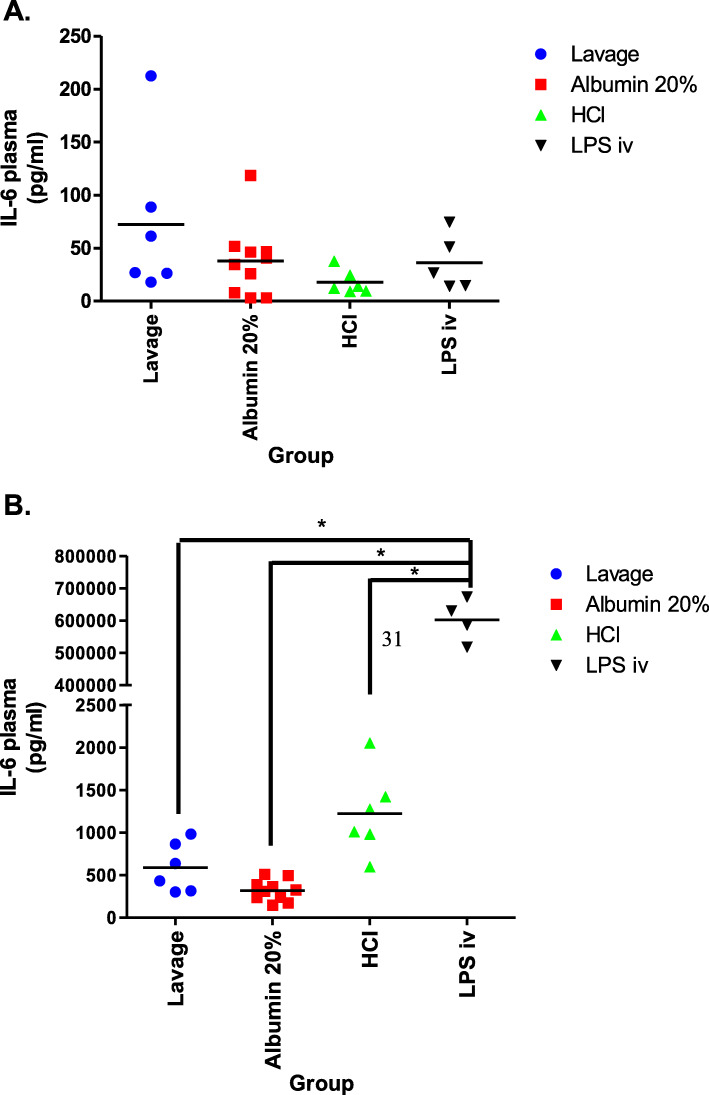
Interleukin-6 (IL-6) concentration in plasma at the time point at which the criteria for ARDs was met = 0 h (**a**) and at the end of the experiment at T = 4 h (**b**). There was no difference in IL-6 concentration between all groups at the beginning (6 A). At the end of the experiment, the IL-6 concentration was significantly higher in the LPS iv group as compared to the other three groups (6 B; * *p* < 0.001). The concentration of IL-6 in plasma was higher in each corresponding group at the end of the experiment (T = 4 h), as compared to the time point at which the criteria for ARDs was met (T = 0 h), but this difference was only significant in the LPS iv group (**a** and **b**; * *p* < 0.001)

The IL-6 concentration in BALF was significantly higher in the LPS iv group as compared to the other three groups at T = 4 h (Fig. 6). The IL-6 concentration in plasma was measured both at the point in time when the ARDS criteria was met, T = 0 h (Fig. 7 A), and at the end of the experiment at T = 4 h (Fig. 7 B). IL-6 concentrations did not differ between the experimental groups at the beginning of the study period (T = 0 h) (Fig. 7 A). The concentration of IL-6 in plasma was, however, higher in each corresponding group at T = 4 h (in comparison to T = 0 h), but this increase was only significant in the LPS iv group (Fig. 7 A and B). At the end of the experiment, the IL-6 concentration was also significantly higher in the LPS iv group when compared to the other three groups (Fig. 7 B).

Histology of the lung showed no significant differences in the mean alveolar size, alveolar wall thickness and in lung injury score (Table 2) between the four groups.

## Discussion

In a comparative study, we tested four different techniques of inducing ARDS in adult ewes. Three models induced ARDs via a pulmonary injury (ARDSp), and one via sepsis due to systemic endotoxemia (ARDSexp). We investigated whether these four models would show different outcomes in terms of respiratory, cardiovascular and inflammatory parameters. The gas exchange (both oxygenation and ventilation) was severely affected with higher OI and lower VEI in the LPS iv group (ARDSexp) and HCl group (ARDSp). The circulation deteriorated in the LPS iv group over the course of the experiment, as reflected by lower diastolic blood pressure. The systemic LPS which induced ARDSexp was so severe that more than one organ system was affected. The LPS iv group had a higher IL-6 concentration in BALF and plasma as compared to the other three groups. These results in the LPS iv group are, however, not completely consistent with the results of the study conducted by Rosenthal et al. [12]. Rosenthal and colleagues used a pig model in which they induced ARDS using different methods. They found that broncho-alveolar hydrochloric acid instillation and lavage caused hypoxemia in pigs, while short term bolus endotoxin infusion did not result in hypoxemia [12]. However, while hypoxemia is a known effect of intravenous installation of LPS, it can take 2–4 h after start of infusion for this effect to become apparent [10, 14]. We therefore included the LPS iv group in the experiment after the ewes had met the criteria of ARDS, taking into account the time interval needed to induce hypoxemia. We standardized the starting time of the 4-h study period as the time point at which hypoxemia occurred, thus the time point at which the animals fulfilled the criteria for ARDS. We showed that inducing ARDS in the LPS iv group (ARDSexp) took significantly longer than it did in the other three groups. As a result, the time of the whole experiment, the time of euthanization, was longer in the LPS iv group. We have no means to assess the effect of this difference on the whole experimental procedure. The effects on the circulation in the LPS iv group in both our study and the study conducted by Rosenthal were comparable and known side-effects of the intravenous LPS model [10, 12, 14]. The LPS iv group represented ARDSexp where the injurious stimuli were not administered intrapulmonary. Despite higher systemic and pulmonary IL-6 concentrations, no differences in histological findings were detected between the LPS iv group and any of the other ARDSp groups, suggesting that the preset definition of ARDS and the short duration of the experiment resulted in a severe lung damage irrespective of the intrapulmonary or extrapulmonary origin of the injury. Taken together, the LPS iv group modeled septic shock with ARDS in a very profound and reproducible manner. The progressive deterioration in the LPS iv group was very different from the course of deterioration in the ARDSp models induced by lung lavage and intratracheal albumin 20%. The OI and VEI improved over time in both groups when ARDS was induced mainly by surfactant removal or inactivation, respectively. Secretion of surfactant or production of surfactant by alveolar cells may have increased the active fraction of the surfactant pool, which may be the reason for this improvement, even while the total surfactant pool was not different.

One of the limitations of our study is the relatively short follow-up period. The focus of our study was the early, acute phase of ARDS. Mortality or long- term effects were beyond the scope of this study. We selected the (female) sheep model as a large animal model that could be used to assess the different models of ARDS and compare them to the pig models outlined earlier. All sheep were of the same gender in order to limit the effects of any potentially confounding variables. In order to mimic the human clinical situation, we did not start the study at the time of inducing ARDS, but rather when all animals had met the criteria for ARDS. Our results are in line with human studies, in which the cytokines were elevated in all forms of ARDS [28, 29]; this supports the value of our models. Nevertheless, it is difficult to translate the results of our study to the human situation. There are many differences between sheep and humans; of particular relevance to this study are the differences in poly-nuclear macrophages in the lung. Assessing these immunological differences was beyond the scope of this study.

## Conclusion

In this study, we found significant differences in OI, VEI, diastolic blood pressure, and IL-6 concentration in BALF and plasma between different models of ARDS in ventilated sheep. All models were potent inducers of ARDS. We did not find injury specific pulmonary changes, but we did observe different clinical developments over the study period of 4 h. On the one hand, clinical deterioration was present in the LPS iv group, on the other hand clinical improvement was observed in the lavage - and albumin groups. We can conclude that the different causes of ARDS resulted in the same clinical starting point but that even in short time experiments the underlying causes affect the clinical properties of the model.

## Data Availability

The datasets used and/or analysed during the current study are available from the corresponding author on reasonable request.

## Abbreviations

ARDS: Acute respiratory distress syndrome
ARDSp: Pulmonary acute respiratory distress syndrome
ARDSexp: Extra-pulmonary acute respiratory distress syndrome
AWT: Alveolar wall thickness
BALF: Broncho-alveolar lung fluid
DSPL: Disaturated phospholipids
FiO2: Oxygen fraction
HCl: Hydrochloric acid
IL-6: Interleukin-6
IL-8: Interleukin-8
LPS: Lipopolysaccharide
MAP: Mean alveolar pressure
MAS: Mean alveolar size
MUMC+: Maastricht University Medical Centre +
NaCl: Sodium chloride
PaCO2: Arterial partial pressure of carbon dioxide
PaO2: Arterial partial pressure of oxygen
PEEP: Positive end expiratory pressure
PCV: Pressure-controlled ventilation
PIP: Positive inspiratory pressure
OI: Oxygenation index
RR: Respiration rate
VEI: Ventilation efficacy index
Vt: Tidal volume
